# Perceived stress among graduate students in health sciences at a military university: a mixed-method approach

**DOI:** 10.1186/s12909-024-06326-w

**Published:** 2024-11-21

**Authors:** Omar Mushtaq, Michael Soh, Ting Dong, Steven J. Durning, John Melton, Khalilah M. McCants, Laura Taylor, Laura Baumann, Bolanle Olapeju

**Affiliations:** 1 Department of Preventive Medicine and Biostatistics, Uniformed Services University, 4301 Jones Bridge Rd, Bethesda, MD 20814 USA; 2 Graduate School, University of Maryland, Baltimore, MD USA; 3 Department of Health Professions Education, Uniformed Services University, Bethesda, MD USA; 4 Daniel K, Inouye Graduate School of Nursing, Uniformed Services University, Bethesda, MD USA; 5 Graduate Education Office, Uniformed Services University, Bethesda, MD USA

**Keywords:** Stress, Graduate students, Military, Active-duty, Coping

## Abstract

**Background:**

There is a gap in the literature about the experiences of active-duty military students pursuing a graduate degree in health sciences at a university that emphasizes a military context. This exploratory study investigates how graduate students navigate stress in the context of a military university.

**Method:**

The study applied a mixed-methods approach including a survey (*N* = 59) and in-depth interviews (*N* = 16) of students enrolled in a graduate program at the School of Medicine (368 students) and School of Nursing (187 students). Survey data was collected via email blasts to various health science departments. In the survey, students could opt-in to complete in-depth interviews. Survey data were analyzed using cross-tabulations while a transcendental phenomenological approach was employed to code and analyze qualitative data.

**Results:**

Survey findings showed that more civilian students (70.6%) felt more noticeably and severely stressed than their active-duty counterparts (64.3%). Active-duty students were more likely to see their grades as a source of stress (76.2%) than their civilian counterparts (64.7%). Active-duty students were less likely to see finances as a source of stress (67.7%) than their civilian counterparts. Active-duty students were not as connected to their families and friends as their civilian counterparts were. The interviews found that active-duty students anticipated the coursework and were adept at juggling multiple demands; whereas, the civilian students centered their concerns around culture and collaboration. Active-duty students saw their program as less of a “big deal;” whereas, civilian students highlighted how they were concerned about the program and financial situations. Active-duty students relied on technical knowledge to understand their well-being; whereas, civilian students understood their well-being in holistic terms. Active-duty students focused on the idea of the individual, and how they take personal responsibility; whereas, civilian students focused more on self-care and compassion.

**Conclusion:**

The study found how civilian and active-duty students experienced stress, stressors, and other aspects that contextualize their experience with stress. Study findings may inform the design of interventions to improve student well-being and resilience mechanisms among similar graduate school contexts.

**Supplementary Information:**

The online version contains supplementary material available at 10.1186/s12909-024-06326-w.

## Background

Literature related to the experiences of active-duty military students pursuing a graduate degree in health sciences is scarce. Military health science institutions emphasize military health care, leadership, and public health. Military students, or students that are on active military duty while they complete their undergraduate/graduate education), may have additional roles or identities that further influence how they experience and/or deal with stress in graduate education. Unlike their civilian counterparts, active-duty military students must undergo military training and possess the necessary skills and resources for successful completion of assigned military-related tasks. Studies suggest that about two-thirds of military medical students experience burnout and they typically cope through exercise, social support, and work-life balance [1]. Prior work has found that military undergraduates respond well to stress and demonstrate resilience [1, 2] presumably due to accountability structures, physical training [2], thought and behavior regulation [3] and strong social ties with their family, unit, and community [3].

While military students have unique experiences with stress, students (civilian and military), navigate multiple individual-, interpersonal- and specific contextual-level stressors when accessing graduate education [4]. Individual-level stressors, or stressors internal to the person, include lack of sleep, age or gender discrimination, financial difficulties [5, 6], and job prospects [7]. Interpersonal-level stressors, or stressors that relate to interpersonal relationships, may arise from contentious relationships between peers, colleagues, and faculty [8–10]. Graduate students also experience weakened ties with friends and loved ones and the loss of their social support systems [11]. Contextual-level stressors, or stressors that arise from one’s immediate environment, are typically related to the degree program or institution and may include academic workload [8, 10], performance expectations [11], and program deadlines [12]. Intense degree programs such as medicine or law are even associated with negative health consequences, such as high levels of anxiety [13], depression [13], suicide ideation [13], and burnout [13, 14]. These stressors can negatively impact students’ graduate education experience, academic performance, and even long-term career satisfaction [14].

This exploratory study investigates how graduate students navigate stress in the context of a military university which provides educational experiences within the military context. Uniformed Services University (USU), located in Bethesda, Maryland USA supports the readiness of America’s Warfighter and the health and well-being of the military community by educating and developing uniformed health professionals, scientists, and leaders; conducting cutting-edge, military-relevant research; and providing operational support to units around the world [15]. The institution educates graduate students in health sciences in the context of the military even though not all students are active-duty service members. Thus, given the literature that reports that military students experience stress and resilience more than their undergraduate counterparts, the primary research question of this study is: “What factors impact civilian and active-duty graduate students at USU?” Understanding how military students perceive and respond to stress patterns is crucial to develop interventions and strategies that optimize students’ graduate education experiences and outcomes. By addressing this question, we will then be able to further explicate on the processes by which students (military and civilian alike) experience stress.

A relevant theoretical framework is the Transactional Model of Stress and Coping [16] developed by Lazaras and Folkman. The model posits that individuals experience stressors, evaluate said stressors, and develop coping mechanisms [16]. When individuals experience stress, they experience a “threat” or “challenge” from either internal or external sources. From these sources, they then evaluate these stressors via the “primary appraisal process.” These threats can then trigger either anxiety or fear, or they are seen as growth opportunities. Next, individuals then respond to the stressor with coping resources and strategies, based on the ability to utilize these resources.

## Methods

### Study context and population

This study specifically focuses on enrolled graduate students’ experiences at USU’s School of Medicine and School of Nursing. USU’s School of Medicine enrolls 368 students and its School of Nursing enroll 187 students respectively. Graduate programs included two-year Masters’, three and four-year doctoral, and three-year terminal anesthesiology degree programs. Masters students are typically active-duty military while civilian students are admitted to the university’s doctoral programs. USU’s Graduate School of Nursing includes the three-year Doctor of Nursing Practice (DNP) and PhD programs. The DNP program admits only military students while Civilians are admitted in the PhD program. USU students are taught in-person by both civilian and military faculty. Eligible participants were adults (aged 18 years or older) currently enrolled in a graduate program. Programs at the School of Medicine include: Tropical Medicine, Environmental Health Sciences, Molecular and Cell Biology, Public Health, Clinical Psychology, Medical Psychology, Neuroscience, Health Administration and Policy, Emerging Infectious Diseases, and Medical Zoology, Leadership, and Health Professions Education. There are seven graduate programs enrolling PhD (to include our MD/PhDs), Masters, and Certificate learners. The vast majority of the programs operate mostly face-to-face with one program being almost fully remote (Health Professions Education). Generally, the PhD programs take around 4–6 years to complete, the Masters programs take around 1–3 years to complete, and the certificates take around 6 months-1 year to complete. About 60% of the students are military and 40% are civilian, with a total enrollment usually around 275 students. Our curriculum is comprehensive and is both clinically and academically rigorous, as it is specifically designed to support the educational needs of the uniformed services” [15].

For the Nursing students [17], the Graduate School of Nursing (Graduate School of Nursing) awards three degrees: Master of Nursing Science (MSN), Doctor of Nursing Practice, and Doctor of Philosophy (Ph.D.) in Nursing as well as a Post-Master’s Certificate. The Masters of Nursing and Doctor of Nursing Practice (DNP) are divided into specialties by the following programs: Adult Gerontology-Clinical Nurse Specialist; Certified Nurse Anesthetist, Family Nurse Practitioner, Psychiatric Mental Health Nurse Practitioner, Women’s Health Nurse Practitioner. The MSN is a two-year program while the DNP is a three-year program. The program is ranked in the top 5% of more than 600 nursing programs nationally, the GSN was designated by the National League for Nursing as a Center of Excellence for Promoting the Pedagogical Expertise of Faculty and Enhancing Student Learning and Professional Development. For two decades the certification pass rate and employment rates were 99% and 100%, respectively [17].

### Data collection

This was a mixed methods study including a quantitative cross-sectional survey and qualitative in-depth interviews to gain insight into how students manage stress. The survey tool provided cursory information about stress while the in-depth interviews provided a more detailed account of the quantitative findings. Both the survey tool and interview instruments were influenced by the Transactional Model of Stress and Coping. Students were recruited into the cross-sectional survey via email blasts which explained the purpose of the study, while stressing that participation was voluntary. The survey lasted about 27 min on average and included validated open and close ended questions on students’ demographics, perceived workload and stress levels, emotional response [18], resilience [19], and their intended duration of stay within the military as applicable (See Supplemental Table 1). A total of 77 students attempted the survey while 59 students completed it for a completion rate of 11%. The overall response rate is 10% (555 eligible currently enrolled graduate students).

**Table 1:** Study respondent demographics by active-duty status

Quantitative survey	Civilian (*N* = 17)	Active-duty (*N* = 42)	Total (*N* = 59)
Male	5 (29.4%)	18 (42.9%)	23 (39.0%)
PhD	17 (100%)	31 (73.8%)	48 (81.3%)
White^a^	13 (76.5%)	30 (71.4%)	43 (72.0%)
Married	4 (23.5%)	29 (69.1%)	33 (55.9%)
In-depth Interviews	Civilian (*N* = 6)	Active-duty (*N* = 10)	Total (*N* = 16)
Male	0	6	6
Female	6	4	10

^a^Total race/ethnicity categories include Asian (*n* = 3), Black/African American (*n* = 7), Hispanic (*n* = 1), White (*n* = 43) and Unanswered (*n* = 5)

At the end of the survey, students were asked to opt-in to an hour-long in-depth interview which provided a deeper exploration of the causes and mitigators of stress within the USU context. The in-depth interviews mapped the context of stress and stressors within the graduate experience, the variety of coping strategies employed by students, military readiness, and potential ways to improve graduate student well-being (See Supplemental Table 1). A total of 16 students were interviewed. Interviews were conducted online through Google Meet, and the interviews were recorded, transcribed, and stored on a secure server. Qualitative data was collected until saturation occurred. This occurred when no new information was able to address the research question, and that the themes were fleshed out until completion [20]. Sample sizes of 6–12 are collected from in-depth interviews because the sample was homogenous [20]. Phenomenology projects usually have a sample size of 5–25 respondents [21].

### Data analysis

Cross tabulations of survey data explored students’ demographics, perceived workload, and stress as well as their coping mechanisms by their military status and degree program (Masters versus Ph.D.). Due to sample size and power considerations, no statistical differences were explored. In-depth interviews were analyzed using transcendental phenomenological methodological framework to understand how particular phenomena occur within a specific context [21]. In transcendental phenomenology, the goal of the project is to reduce information down to significant statements and quotes to find the essential insights and provide a textual description of respondents’ overall experience [20].

In terms of the steps that we followed to analyze data, we conducted a phenomenological analysis [22]. We engaged in phenomenological reflection in order to bracket our experiences based on phenomenological principles and assigned preliminary codes systematically until they were categorized into themes [20].We reflected on our own experiences and perceptions of the situation and intentionally set them aside before analyses [22]. We then sought specific statements that represented specific experiences via horizontalization [22]. The statements were then grouped into themes based particular meaning units. We then provided a composite description of the essence of how students experienced stress [22]. Once these key concepts were identified, they were sorted into common themes.

 As part of reflexivity, the study authors acknowledge insider and outsider biases that could influence the data collection. Data collection and analyses were primarily conducted by a postdoctoral researcher with little familiarity with USU students and their environment to offset any insider biases. However, the postdoctoral researcher was an institutional outsider, who did not have experience navigating the department, but had eleven years of qualitative research experience. Other authors were faculty and administration at the university who taught and conducted research at the university. The postdoctoral researcher worked in tandem with the other co-authors in the qualitative data analysis process.

Often, member checking has been used to verify the veracity of experiences in research [23]. However, we did not conduct this due to the fact that students were not available by the time the data had been analyzed. To address the notion of defining expertise [24], two researchers had extensive experience working in Student Affairs and Administration, and one was trusted by students since she was specifically mentioned in interviews. To ensure clarification, we asked probing questions, paragraphs of information to allow respondents to confirm or deny information, and asked open-ended questions during the interview process [25].

### Ethical considerations

This study was reviewed and approved for execution by the USU Human Research Protection Program Office (Reference No. 957211). Given that the study examined mental health and researched potentially sensitive subjects, students were given available supporting resources for their reference or use. In terms of confidentiality, interviews were stored on a secure server backed by government security measures including authentication and other firewall software. All participants provided written informed consent in the survey and in-depth interviews. The study uses pseudonyms to protect the identity of the respondents and maintain confidentiality.

## Results

### Description of study population

A total of 59 students were surveyed while 16 students participated in in-depth interviews (Table 1). Overall, study respondents were more likely to be active-duty military students compared to civilians.

### Quantitative survey results

Table 2 summarizes the quantitative results related to students’ i) graduate education context, ii) perceived levels of stress and ii) factors influencing stress during their graduate education.

**Table 2:** Survey respondents’ context and stress by active-duty status

	Civilian (*N* = 17)	Active-duty (*N* = 42)	Total (*N* = 59)
**Graduate Education Context**			
Spends over 40 h a week on a graduate degree	13 (76.5%)	34 (81.0%)	47 (80.7%)
Describes themselves as overworked	4(23.5%)	12 (28.6%)	16 (27.1%)
Average percent of time spent on specific activities			
Attending classes	20.6%	30.2%	27.4%
Assignments	17.0%	28.2%	25.0%
Research	54.2%	27.1%	34.9%
Other activities	8.2%	12.1%	11.0%
**Perceived Stress**			
Felt noticeably/ severely stressed this academic year	12 (70.6%)	27 (64.3%)	39 (66.1%)
**Factors Influencing Stress**			
Studies or grades	11 (64.7%)	32 (76.2%)	43 (72.9%)
Financial pressures	11 (64.7%)	11 (26.2%)	22 (37.3%)
Family issues	7 (41.2%)	19 (45.2%)	22 (44.1%)
Work or military-related issues	9 (52.9%)	20 (47.6%)	29 (49.2%)
Health	9 (52.9%)	9 (21.4%)	18 (30.5%)
Relationship with (some) faculty	5 (29.4%)	6 (26.2%)	16 (27.1%)

#### Graduate education context

The majority (80.7%) of both active-duty and civilian students surveyed spend over 40 h a week on their graduate degree, only less than a third (27.1%) described themselves as overworked. Students also detailed the breakdown of their work week in terms of the percent of their time used for specific activities. On average, students spent about 35% of their time on their research, and about a quarter of their time attending classes (27%) or doing assignments (25%).

#### Perceived stress

About two-thirds (66.1%) of all students noted that they felt noticeably or severely stressed during the academic year. Higher proportions of civilian (70.6%) compared to active-duty (64.3%) students felt noticeably or severely stressed.

#### Factors influencing stress

Key factors influencing students’ stress levels included their studies or grades (72.9%), work or military-related issues (49.2%), and family issues (44.1%). In addition, higher proportions of civilian students noted financial and health issues while more active-duty students noted work or military-related issues.

### Qualitative in-depth interview results

In-depth interview findings provide further elucidation on students’ i) graduate education context, ii) perceived levels of stress and ii) factors influencing stress during their graduate education.

#### Graduate education context

The graduate education context is described using the following themes: Students’ perspectives of i) their pre-enrollment expectations and ii) current impressions of their program.

##### Pre-enrollment expectations

Interviews suggested that civilian students expected challenges and demands on their time. They used descriptors like “hard” and “intense” to describe their expectations of their graduate program. Civilian students expected that their schedules would revolve around a constant flow of work and compared going to school to working a full-time job.

“[My] expectations were definitely that it would be a lot of work. It would be pretty hard, you know”

(Jasmine Olivine, PhD Student, Civilian).

“My expectations of the workload was 24/7. I probably would be working all the time. I was aware of the kind of true stereotype of students working themselves to death. So I had fully expected working during the week and also on the weekends and things like that and just not having too many boundaries around what my workload would be”

(Misty Cerulean, PhD Student, Civilian).

Similarly, active-duty students also anticipated a high workload upon entering graduate school. They believed that they would manage multiple tasks such as writing, reading, studying, and orating multiple presentations. They were used to the rigidity and structure of military life and they did not anticipate their graduate education experience would be problematic. They assumed that the faculty were the “experts” and the authority figures. They also assumed that because they were focusing on coursework, they would not have the demands that typical military service members would have, and that their time could be used as a reprieve,

“Because I was doing the Army for the last four years, and for me, like anything else would just be easier. When I expected to be able to hold over my time, which is a bonus, and [I did] not have to deal with the stressors that the Army brings up. So, like everything that I’m doing now is way more manageable than while I was doing actual military things”

(Karen Johoto, PhD Student, Active-Duty).

Notably, while the active duty students assumed they would have hectic schedules, some of them based this on previous graduate school experience:

“Having been to graduate school prior, I had a pretty good idea of what the expectations were and what the workload was going to be during USU. Doing it [classes] in person, I expected the workload to be quite a bit higher just because you’re not only doing the reading and the writing requirements, but you’re also listening to the lectures and conducting yourself on practicum and things like that”

(Rodney Ketchum, PhD Student, Active Duty).

Active-duty students did not expect to be concerned about their military assignment because they were receiving education as part of their assignments. While they noted administrative factors, readiness, and physical training tests that intersected with their military experiences, they were able to focus on their graduate school experiences. A few active-duty students, however, noted that being in the military was more defined compared to being in graduate school.

“[Balancing military obligations with graduate school is] easy because this is my duty assignment. You know, they say you’re gonna go to school for two years so I show up in uniform and go to school. So it’s extremely easy. You gotta maintain your medical readiness. So I have to go to the dentist and have to do my physical. I have to do my physical fitness test, but I mean those things are easy here at USU. It’s extremely easy to be a service member and full-time school at the same time because school is my assignment”

(Blaine Cinnabar, Master’s Student, Active-Duty).

##### Current impression of graduate program

Civilian students focused more on the culture of the program rather than its difficulty. Some noted student collaboration and faculty support as pluses for their program. Some also noted that they received individualized attention from faculty as opposed to seeing students as a group. Other students noted the lack of communication within their department which they felt was unexpected.

“I think some of them [expectations] matched up, but the big one was that I really relied on my peers a lot more than I expected. [In] the first couple years in classes, they were really important to my success. Basically, we worked together and we supported each other studying for hours every single day together. I never thought of myself as a group study person then grad school certainly changed that”

(Whitney Goldenrod, PhD Student, Civilian).

Active-duty students largely described that their program met, or, in some cases, exceeded their expectations, also noting student collaboration and faculty support. Some mentioned that their program could have had a stronger military focus while others noted that some of their peers were impacted by a rigorous routine and financial difficulties.

“All of my expectations were met or exceeded between the collaboration of my peers, and then the faculty have been insanely incredible. The only thing that didn’t meet my expectations, which is a good thing, is that it’s not as military focused as I thought it would be; whereas, I was expecting something like an Air Force Academy where everything is very structured and very focused on the military. This is more of a graduate university that has an emphasis in military studies which I think is an incredible way to do it”

(Rodney Ketchum, PhD Student, Active-Duty).

#### Perceived stress

Students first described their understanding of well-being and stress before discussing their perceived stress during their graduate education.

##### Understanding of well-being and stress

Civilian students described well-being as “mental, physical, and emotional” health and drew this concept from a technical understanding of how well-being ought to be maintained. Some students described that their ability to manage stress also factored into how they defined well-being.

“Yeah, I think I would define it as “physical and mental health” which I also know can be vague in terms as well. So, I think each individual sort of defines what health is for them in their own way, but I understand well-being is really both of those components together”

(Jasmine Olivine, PhD Student, Civilian).

Some civilian students mentioned the notion of “friends” when describing their ideas of well-being. They also emphasized various leisure and physical activities.

“Examples [of wellbeing] would be taking the time to do things for yourself - both productive and unproductive things [like] doing your household chores[,] going to the gym, but also, seeing your friends and taking a break. I think the key to that is also vocalizing to the people that support you when you need help”

(Erika Celadon, PhD student, Civilian).

Active-duty students also described overall “balance” between various aspects of health including mental, physical, and spiritual types of well-being. Some included “faith” as part of well-being. In particular, active-duty students also highlighted the importance of sleep as a strong component of well-being. Some also spoke about how well-being must be maintained long-term or “longitudinally”.

“It’s a whole body concept so like physically, mentally, spiritually. You can be good, physically, but can you maintain that? I think maintaining those over a long period of time too”

(Janine Fuschia, PhD Student, Active Duty).

Civilian students described the physiological and emotional manifestations of stress. Civilian students defined stress as feeling “uneasy” with “physiological consequences.” For another student, stress was described as “something irritating.”

“Um, stress for me comes in a lot of different ways and forms. For me, it can be physical manifestations where I can feel the butterflies in my stomach. My heart rate [increases]. That’s generally a couple days before a big presentation. That’s stress. And then there’s, like, overall prolonged periods of stress where I’m planning experiments, trying to collect data or preparing for a presentation, that’s, maybe a couple weeks out”

(Whitney Goldenrod, PhD Student, Civilian).

On the other hand, active-duty students associated the term “stress” with the idea of “being overwhelmed.” Some students mentioned that stress existed outside or within “normal” bounds while others mentioned that stress can be “controlled” or “uncontrolled.” One student also mentioned how the environment factored into stress and described stress as a result of specific stimuli, providing examples of stressful situations.

“I think stress is when you feel overwhelmed when you take on more than you should cause sometimes there are going to be challenging times. When you may have multiple assignments or have lots of readings, those times are going to be there. So, I think stress is when you don’t know how to handle them effectively. Yeah. So that’s how it contrasts when you have more on you than what you don’t know”

(Blaine Cinnabar, Masters Student, Active-duty).

#### Factors influencing stress

In the interviews, students reflected on the role of their program, personal responsibilities, family, friends, and coworkers on their experience of stress.

##### Program

Civilian students noted that the program “significantly” impacted their stress. This was because some did not see the connection between various aspects of the curriculum or perceived clear communication about various processes within the program. Some students perceived this lack of communication as “confusion” which was distressing and exacerbated their stress.

“[The program causes stress] significantly because it’s a very small program, and not really feeling like there’s a connectedness or a mentorship within the program. And like I said earlier, the mismatch of like. what you think you taught me and then what you tested me on. There’s kind of that mismatching and not a lot of attempts to kind of rectify that.”

(Agatha Indigo, PhD Student, Civilian)

To an extent, civilian students also described the program’s culture, including notions of lifestyle factors. For example, some students described how an ethos of “not being able to work hard enough” and losing sleep impacted their experiences with the program. Some civilian students described the workload specifically as something that factored into how they experienced stress.

“I think graduate student culture is probably the big one. I think there’s this weird perception that students need to be overworking themselves to such an extreme. If you’re not sleeping two hours a day, and you’re sleeping more, that’s ridiculous. You’re not working hard enough. There’s this over glorification of that. Just like being an overachiever and overworking yourself, that contributes to my stress.”

(Erika Celadon, PhD Student, Civilian).

Active-duty students largely did not perceive their program as stressful, but, rather, were either indifferent to stressors and did not see them as a “big deal.” They largely accepted the workload and different aspects of the program, specifically, noting that the program offered different avenues of support to its students, including the availability of supportive resources, students, and faculty.

“Um, they [the program] didn’t really add any stress. You know, my classmates? We get along really well. So, we support each other. We encourage each other. You know, if somebody’s having an issue with any particular assignment or a topic, we kind of come together or give examples, or we may help them out with that topic. Give some additional support or, you know, try and help them understand whatever that concept is. So they don’t necessarily add any stress. [They] definitely help reduce stress if necessary because that’s a group of folks you can definitely go to.”

(Blaine Cinnabar, Master’s Student, Active-Duty

##### Personal responsibilities

Civilian students reported that their family and home responsibilities were other obligations. This included partners, parents, children, the elderly, and other familial responsibilities, such as finances. However, other civilian students noted the general stressors associated with being a graduate student and balancing that responsibility with extracurricular activities.

“I have my children, my household, aging parents, you know, finances that cross all of that”

Agatha Indigo (PhD Student, Civilian).

Civilians used words like “a lot” and “significantly” to describe how these obligations impacted their daily experiences. Some civilian students considered the positive and negative aspects of how these obligations impacted their experiences, but most of the civilian students underscored the importance of having to navigate stressors in their lives, including diminished finances.

“So a lot of these other obligations can turn financial, especially since we’re not really able to take another job or anything. That income/financial stress can kind of impact my ability to sit down and do work because it’s either ‘I do this graduate student work, or fulfill my other obligations’ but, also, trying to balance everything because my primary income is coming from my stipend”

Misty Cerulean (PhD Student, Civilian).

On the other hand, active-duty students did not feel stressed by their finances.

*Um, I don’t think it’s [finances] really a factor because school is free. So I’m not trying to pay off student loans. I’m getting my same military check. So, not really a stressor managing money and budgeting like normal.”*

(Blaine Cinnabar, Master’s Student, Active-Duty)

##### Family

Civilian students saw the family as mostly supportive. They usually used the words “supportive” and “understanding” to characterize their relationships with their families. Some students were able to communicate their concerns, and “vocalize” their concerns, with their family. Others reported that their family members gave them strength. Some civilian students, however, mentioned that their families negatively impacted their lives.

“I think they’re [families are] helpful. They try to be helpful there. Their lives are different than mine. So it’s not a direct relatability. There’s not a lot of direct relatability. The friends that I have at school definitely can relate and understand because they’re going through the same thing. But the rest of them are either they’ve not gone to graduate school, or they’re so far removed from it. It’s maybe hard for them to imagine trying to do it as a mom.”

(Agatha Indigo, PhD Student, Civilian)

In contrast, several active-duty students saw their families as external obligations. Some reported that they were parents and that they did not have time for themselves because of family. Others reported that family significantly impacted their time away from school and struggled to maintain their time. Some were able to prioritize family and relented on connecting with them.

* “So yeah, just being the mom of young kids because they need me all the time and you know. When I go home, it's not my time. It's not for me. I can't just go home, study, and relax. There's no such thing. Even on the weekends. so that is a big factor in terms of stress in daily life.”*

##### Friends

Many civilians reported that friends were also seen as supportive because they experienced similar lifestyles. Interestingly, some noted that they did not have relationships with others outside their work, so their coworkers became their friends, while others mentioned they did not have coworkers so their friends were largely their peers. Civilian students saw their friends as sources of support, and they also largely understood how civilian students experienced stress. Friends did not exacerbate stressors.

“A lot of my friends actually work a lot too so I guess it’s really similar in that sense in that I don’t do a lot with friends because I don’t have a whole lot of time to do stuff with them, and they also don’t have a lot of time either. So, like, we don’t do a lot together but it is very, um, distressing. I guess to do things with it, if it’s just like, let’s get coffee.”

(Loreli Lapras, PhD Student, Civilian)

Many active-duty students did not prioritize their families, and, consequently, were unable to maintain their friendships, some active-duty students identified their significant other/partner as “their friend” and some differentiated their peers as “classmates.” In other words, active-duty students conflated their familial ties with friendship. Some active-duty students perceived their friends as supportive, but some also reported that their friends did not understand their workload.

“Um, well, I’m 40 years old so my wife was my best friend. So I guess I really don’t like hanging out with other people. As far as friends, I mean I have my classmates, but, you know, we support each other. We have fun. We talk about upcoming assignments. We support each other as far as studying; so, other than that, I don’t really have a huge friend support network. You know, it’s really my wife[,]my brother[,] and parents.”

(Blaine Cinnabar, Master’s Student, Active-Duty Student)

##### Coworkers

For several civilian students, coworkers were either lab mates or their colleagues in internships. Civilian students reiterated that workers were largely lab-mates and that they generally saw their “coworkers” as helpful peers. They also noted that they reported that their coworkers ameliorated their stress.

*I think like my class, like the people in my lab*. *They always are very helpful because whenever I’m stressed [or if] I don’t know how to do something. I can ask them, ‘How do I do this? What should I do?’ Not that I’ve gone to them for advice so many times, [but] I think about lab-related stuff like this, and they’re always really helpful. I guess I kind of consider the students co-workers but like some of the other people in other labs, that kind of thing.*

(Lorelei Lapras, PhD Student, Civilian)

Many active-duty students described coworkers as classmates or peers by noting that they were “friends.” In this situation, “coworkers” minimized stress, especially when stress largely came from military administration. However, this was not necessarily the case as a minority of active-duty students recounted experiences where their classmates exacerbated certain stressors often due to a lack of teamwork and transparency.

“Coworkers would be the same as friends in this instance. They were undergoing the same thing I was and talking to them about it made me and them realize that we were all kind of having the same issues which helped lessen the impact. But we were all still feeling the stress from that specific situation”

(Rodney Ketchum, PhD Student, Active-Duty).

## Discussion

Our research highlights potential similarities and differences in how military service members and their civilian counterparts experience stress in graduate education. All students spent copious amounts of time working on their graduate degrees; however, they would not describe themselves as overworked. Active-duty students were stressed with work or military-related activities. However, they anticipated higher workloads and often appeared to naturalize their experiences with the workload, likening their experiences to being in the military and naturalizing the hierarchies of student life. Civilian students on the other hand were concerned about the program and their financial situations, describing their graduate education from a wellbeing perspective that was impacted by mostly academic, personal and family level factors.

The Transactional Model of Stress and Coping [16] provides a theoretical model to explain how active-duty and civilian students experience stress. According to the model, both active-duty and civilian students deal with both internal and external stressors; they evaluate whether or not these stressors are “threats” or “challenges;” and then they apply coping mechanisms to their stressful experiences. However, active-duty and civilian students in our study appeared to experience and interpret stressors differently. As noted, active-duty graduate students typically neutralized stressors, did not have ad hoc psychological responses to stress, and saw stressors as “challenges.” They often emphasized the notion of “self-management” and “personal growth” even though they typically mentioned that the lack of transparency in the workload [5] impacted their experiences. However, in the interviews, active-duty students often saw their families as a source of bad stress because the married respondents planned their life around their families and other active-duty respondents saw their families as interruptions so this finding conflicted with other literature, [1, 3] and did not see a need to marshal resources to cope. Civilian students, conversely, often perceived their experiences as stressful by typically interpreting stressors as threats, marshaling coping mechanisms such as self-care to address these threats [26–28].

These findings present both practical and research implications for future interventions or additional studies. For example, if demonstrated in larger studies, institutional arrangements, and program structures could be stratified to address both civilian and active-duty service members’ needs. Likewise, it is important to consider the timing of data collection where students might have been busy during peak times of data collection. These differences in findings could also exist between other relevant groups such as under-represented learners.

Relevant authorities or mechanisms such as the services provided by Student Affairs should be mindful of collaborative versus hierarchical learning approaches and work styles and offer opportunities for active-duty service member students to thrive. For example, in the findings, active-duty students tended to favor more hierarchical models of instruction where the professor led the class through lectures as opposed to allowing students to produce knowledge so future programs could be tailored to engage active-duty students on a collaborative level. Furthermore, given their itinerant background, ADSM may be presumed to socially isolate themselves from support mechanisms, and they may require additional support outside their immediate families and networks, including other associations and peer groups. Pre-doctoral preparatory programs can help manage expectations, instill preparation for upcoming curricula, and encourage flexible views, particularly among civilian students.

While the scope of this paper focuses on civilian and active-duty population, there are also broader implications on how students at civilian universities in general experience stress and how various factors impact their experience. This study has unearthed how students experience stress and the contextual factors of this stress. Thus, other universities can take note of how multiple avenues, such as funding and support mechanisms can possibility mitigate stress in non-military contexts and improve curriculum development in these contexts.

Additional research should explore specific avenues to improve student satisfaction and lower stress. For example, students reported spending over 40 h a week on their problem but not feeling overworked. Thus, it would be important to understand the myriad factors, such as possible student support services, affinity groups, collaboration opportunities, networking events, mentorship programs, student evaluations of programs and tenure-track faculty, and other factors that would contribute to a positive graduate program environment. Such research might need to be stratified by ADSM status to uncover specific insights that would inform the design of relevant interventions.

A key limitation is the study’s ability to generalize findings because of its exploratory nature, low survey response rate, lack of member checking and limited sample size. However, future research can focus on larger sample sizes. We also note that various aspects of the different programs might have impacted the students’ stress, but, in this analysis, we centered our research methodology around a phenomenological framework which limits the data analysis to common experiences. Future studies can expand upon the nuances of these programs with other qualitative methodologies, such as grounded theory. This study was conducted in a military university so might not indicate how typical civilian students in civilian universities respond to stress. Likewise, collecting sensitive information about mental health could have impacted data collection and response rate.

The study also provided insights about how students navigated stress, but did not investigate the environment in which the students navigated. Also, students made assumptions about the nature of hierarchy within their program. In both these cases, an ethnographic evaluation would be important to understand how active-duty and civilian students contextualize meaning. Furthermore, there might have also been temporal issues where the specific timeframe in the academic calendar when the data was collected could have impacted students’ discernment of their experiences of stress. Also, each graduate program varies in terms of its length of time, curriculum, and rigor. While these impact student experience we focused on the graduate experience, future research can investigate specific ways that the unique program can impact student experiences.

This mixed method study explored differences in how active-duty and civilian students navigate graduate programs based in a university that is centered around military goals. The study uncovered potential differences in perceptions regarding stress, stressors, and factors influencing active-duty and civilian students’ ability to handle stress that should be explored further in future studies. Study findings can inform the design of interventions to improve student well-being and resilience mechanisms among similar graduate school contexts.

## Supplementary Information

Supplementary Material 1.

## Data Availability

The datasets used and/or analyzed during the current study are available from the corresponding author upon reasonable request.

## Abbreviations

ADSM: Active-duty Service Members
DNP: Doctor of Nursing Practice
GSN: Graduate School of Nursing
HRPPO: Human Research Protection Program Office
MNS: Master of Nursing Science
PhD: Doctor of Philosophy
USU: Uniformed Services University
