# Supplementary material for: Perceived stress among graduate students in health sciences at a military university: a mixed-method approach
Source: BMC Med Educ. 2024 Nov 21;24:1343. doi: 10.1186/s12909-024-06326-w (PMC11583747; doi:10.1186/s12909-024-06326-w)
Supplement: Supplementary file 1 — Supplementary Material 1. [file 12909_2024_6326_MOESM1_ESM.docx]

| **Supplemental Table 1: Frequencies of Select Survey Questions** | | |
| --- | --- | --- |
| Item | Responses | Total  59 (100%) |
| Which of these currently best describes your workload? | Overworked  Managing  Underworked  Don’t wish to answer | 16 (27%)  41 (69%)  1 (2%)  1 (2) |
| On a scale of 1 to 5, How stressed do you feel on a daily basis during the academic year? | Not stressed at all  Minimally stressed  Manageably stressed  Noticeably stressed  Severely stressed | 0 (0%)  2 (4%)  18 (30%)  29 (49%)  10 (17%) |
| What are the current causes of stress in your life as a graduate student in USU? Select all that apply | Studies/grades  Financial pressures  Family issues  Friends issues  Military related issues  Issues with the significant other (partner)  Work (job-related) issues  Health Related Issues  Sports / Athletics activities issues  My involvement in clubs and organizations  Campus/social life  Relationship with (some) faculty members  Other (specify)  None of the above | 43 (73%)  22 (37%)  26 (44%)  4 (6%)  14 (24%)  8 (14%)  19 (32%)  18 (31%)  5 (8%)  6 (10%)  6 (10%)  16 (27%)  10 (17%)  0 (0%) |
| How has your graduate studies impacted your military readiness? Would you describe the impact in your own words | [Open Text] | N/A |
| How likely are you to remain in the military immediately after your term of service? (N=42 military students) | Very likely  Somewhat likely  Somewhat unlikely  Very unlikely  Don’t wish to answer | 27 (64%)  8 (19%)  2 (5%)  4 (10%)  1 (2%) |
| How likely are you to remain in the military till retirement? (N=42 military students) | Very likely  Somewhat likely  Somewhat unlikely  Very unlikely  Don’t wish to answer | 25 (60%)  10 (24%)  2 (5%)  4 (10%)  1 (2%) |

| **Supplemental Table 3: Select In-depth Interview Questions** | |
| --- | --- |
| Section 1: Overview of graduate school | What got you interested in going to graduate school? Why did you choose the graduate program at USHS? What were some of your expectations of the graduate program at USHS? What were your expectations of the workload in graduate school? What were your expectations of the culture in graduate school? What were your expectations of your faculty in graduate school? What were your expectations of how your peers would support you? How did you expect to balance your military service with graduate school? (if applicable)  Can you walk me through a typical day as a graduate student at USUHS? How did these expectations compare to what you experienced in graduate school? |
| Section 2: Well being | How would you define “wellbeing” in general?  Can you provide examples of your idea of wellbeing as a graduate student at USUHS?  Can you give examples of what you have done to improve your wellbeing recently? |
| Section 3: Stress | How do you define “stress”? How have you experienced this stress in graduate school?  (probe for situation, personal impact, relational factors) Please describe other factors that might have affected your stress level?  Probe: What was happening on a personal level? How did your family affect this event? How did your friends impact this stress? [add graduate support] How did your coworkers impact this stress? How did your program impact this stress? How did this stress resolve? To what extent are you able to apply any lessons learned from this situation?  What factors have contributed to the stress you deal with as a graduate student?  What other obligations do you balance when in graduate school?  How do these obligations impact your ability to be a graduate student?  To what extent do other factors affect your ability to study in graduate school? E.g. health, finances, social support |
